# Awareness of Cancer-Related Malnutrition and Its Management: Analysis of the Results From a Survey Conducted Among Medical Oncologists

**DOI:** 10.3389/fonc.2021.682999

**Published:** 2021-05-13

**Authors:** Maurizio Muscaritoli, Emanuele Corsaro, Alessio Molfino

**Affiliations:** ^1^ Department of Translational and Precision Medicine, Sapienza University of Rome, Rome, Italy; ^2^ Medi-Pragma S.r.l., Rome, Italy

**Keywords:** cancer, malnutrition, cachexia, medical oncologists, awareness, multidisciplinary team, survey

## Abstract

Cancer is a global major public health problem, particularly in Western countries, where it represents the second leading cause of death after cardiovascular disease. Malnutrition is common in cancer patients and differs from starvation-related malnutrition, as it results from a combination of anorexia and metabolic dysregulation, caused by the tumor itself or by its treatment, and causing cachexia. Cancer-associated malnutrition can lead to several negative consequences, including poor prognosis, reduced survival, increased therapy toxicity, reduced tolerance and compliance to treatments, and diminished response to antineoplastic drugs. Guidelines issued by the Ministry of Health in 2017, the most recent ESPEN guidelines and the PreMiO study highlighted an inadequate nutritional support in cancer patients since their first visit, and recommended an optimization of the quality of life of cancer patients in each stage of the disease, also through specific nutritional interventions by multidisciplinary teams. Based on the evidences summarized above, a survey has been carried out on a sample of 300 Italian hospital medical oncologists to evaluate their level of awareness and perception of cancer-related malnutrition and their proposals to implement effective strategies to improve nutritional care in the setting of hospital oncology departments in Italy. The survey results indicate that, despite high levels of awareness among Italian oncologists, malnutrition in cancer patients remains, at least in part, an unmet medical need, and additional efforts are necessary in terms of increased training and hiring of personnel, and of creation of organizational pathways aimed at treatment optimization based on available evidences.

## Introduction

The term “malnutrition” is generically used to include both over-nutrition (leading to overweight and obesity, which are recognized risk factors for onset and progression of hormone-dependent endometrial and breast cancer tumors) (1), as well as under-nutrition and nutritional risk. Malnutrition is commonly associated with disease and can affect all age groups. Cancer-associated malnutrition differs from starvation-related malnutrition, as it results from a combination of anorexia and metabolic dysregulation, caused by the tumor itself or by its treatment. Cancer-associated malnutrition can lead to cachexia, a multifactorial syndrome frequent in chronic diseases and now considered as a comorbidity of cancer, characterized by severe, involuntary loss of skeletal muscle mass, with or without loss of fat mass, increased systemic inflammatory response (2) and increased protein catabolism (3, 4). The agreed diagnostic criterion for cachexia is a weight loss greater than 5% (5). Cachexia by itself may account for up to 20% of cancer deaths (6), and the progressive loss of muscle mass in cancer has been identified in particular as an independent and significant predictor of overall survival (7). Cancer cachexia is currently classified into three stages, namely pre-cachexia, cachexia, and refractory cachexia (5).

If untreated, cancer chachexia leads to a progressive functional loss, poor quality of life, chemotherapy-related toxicity, diminished response to antineoplastic treatments, and poor survival (8). Interestingly, studies carried out in murine models of cancer cachexia showed that the reversal of muscle loss can lead to a prolonged survival, even without inhibition of tumor growth, reduction of fat loss and production of proinflammatory cytokines (9). These data support the maintenance of muscle mass as a therapeutic target to improve survival of cancer patients. Cachexia cannot be fully reversed by a conventional nutritional support, but it is important to establish a continuous treatment schedule based on the implementation of nutrition, food supplements, drug therapy, exercise, and psychological counseling. The anticachectic treatments play a key role in cases of reversible cancer cachexia where, if correctly associated with effective anticancer therapies, they can contribute to preserve the patients’ quality of life and improve the prognosis (10). Conversely, in case of refractory cachexia, defined by a cancer disease not responsive to antitumoral treatment, with low performance status and life expectancy lower than 3 months, artificial nutrition can be integrated into a palliative care program, although, in this phase, the benefits on the cachectic phenotype, if any, are expected to be very limited (11).

Despite the evidences summarized above, three global surveys recently carried out to gain insights on the awareness and treatment of cancer cachexia among healthcare professionals revealed an inadequate management of this comorbidity, as 48% of the participants to the surveys would wait for a weight loss ≥15% to diagnose cancer cachexia and prescribe a treatment, and 61% to 77% of cancer patients did not receive any medication for cancer cachexia before reaching a stage IV disease (12).

The Prevalence of Malnutrition in Oncology (PreMiO), a cross-sectional, observational study (13) involving almost 2,000 patients in 22 sites in Italy, revealed that 51.1% of treatment-naïve patients at their first visit to a medical oncology center were already affected by a nutritional impairment, including risk for malnutrition (43%) and overt malnutrition (9%). Poor appetite was present in over 40% of cancer patients, with variable severity scores depending on the tumor type and stage of the disease, and ascribed mainly to early satiety, taste changes, and nausea.

The evidence-based guidelines on nutrition in cancer research issued in 2017 by the European Society for Clinical Nutrition and Metabolism (ESPEN) (14) provide a series of recommendations for prevention, identification, and treatment of malnutrition in adult cancer patients. In particular, members of the guideline group recommended to screen regularly the cancer patients for the risk or presence of malnutrition.

In addition, the Guidelines for nutritional pathways in cancer patients (15) issued in 2017 by the Italian Ministry of Health (MoH) in collaboration with the joint Working Group of the Italian Association of Medical Oncology, the Italian Society of Artificial Nutrition and metabolism, and the Italian Federation of Volunteer-based Cancer Organizations, recommend an optimization of the quality of life of cancer patients in each stage of the disease, also through the definition by a multidisciplinary team of a specific nutritional plan, type and timing of supplementation, instrumental and clinical/laboratory evaluations of patients’ nutritional status.

Both the guidelines and the above mentioned studies highlight and address the inadequate nutritional support in cancer patients, often since their first visit, and with consequences for adherence, response, and tolerance to cancer therapy, as well as for disease progression and prognosis. A multimodal approach, namely the T.A.R.G.E.T. approach, encompassing both active actions and further research within different domains, namely Teaching, Awareness, Recognition, Genetics, Exercise/Early intervention, and Treatment of cancer cachexia, has been proposed to counteract the development of cancer cachexia (16). Recently, a review by Solheim TS et al. also emphasized the importance of early intervention through a combination of optimal nutrition and exercise in a multimodal approach to weight loss and loss of function in cancer patients (17). Screening represents the first step for early detection and effective treatment of cancer cachexia. Incorporation of the nutritional status evaluation and monitoring should therefore be regarded as a hallmark of good clinical practice in cancer treatment (18). In the context of early diagnosis and prevention of malnutrition in cancer patients, a parallel pathway approach, where oncology and clinical nutrition work in synergy since the beginning of the natural history of the disease and during its whole development, has been proposed to offer to cancer patients a chance to prevent/delay cancer cachexia onset (3).

In this setting, a survey has now been carried out on a sample of 300 Italian hospital medical oncologists to: a) evaluate their level of awareness of cancer-related malnutrition; b) assess the level of implementation of MoH Guidelines for nutritional pathways in cancer patients (15), ESPEN guidelines on nutrition in cancer patients (14) and indications from the PreMiO study (13) in the hospital departments where the oncologists work and, in case, how guidelines affect their clinical practice; c) explore the notions associated with the parallel nutritional-metabolic pathway for cancer patients; d) establish potential strategies to increase the awareness of cancer-related malnutrition among hospital medical oncologists and oncology departments in Italy.

## METHODS

### Survey Design

A 44-item purpose-designed survey has been developed and carried out by Medi-Pragma S.r.l. (Rome, Italy) for 45 days in June 2019 on a sample of 300 active hospital medical oncologists, representative of a reference population of 5,616 oncologists, implying a sampling error between 7.8% and 8% (z score: 1.96; CI: 95%; standard deviation: 0.5).

Informed consent was obtained through a participant information sheet before starting the survey.

Based on the inclusion criteria, participants were hospital medical oncologists, treating patients affected by gastrointestinal tract tumors, pancreatic tumors and head and neck tumors, and at risk of malnutrition during chemotherapy/radiotherapy.

The questionnaire administered to oncologists was semi-structured to meet the objectives of the survey and included questions on participant demographics and questions relating to three key areas: 1) awareness and understanding of malnutrition; 2) attitude and propensity of clinicians and their centers towards clinical nutrition; 3) suggestions on how to increase the awareness of physicians about the importance of clinical nutrition in the oncological setting (**Table 1**).

**Table 1:** Questionnaire items with answers, expressed as absolute values or as percentages, when indicated.

QUESTIONNAIRE ITEMS	
INTRODUCTION: SCREENING	
**Q1. What is your specialty?**	
a. Oncology	100%
b. Other (quit the survey)	0%
**Q2. Where do you work?**	
a. Private hospital	6%
b. Public hospital	79%
c. Teaching hospital	15%
d. Other (quit the survey)	0%
**Q3. How many cancer patients did you visit approximately in the last month?**	
Number of patients (mean ± SD):	137 ± 89.3
**Q4: What kind of tumor do you mainly focus on?**	
a. Solid tumors	84%
b. Liquid tumors (if 100%, quit the survey)	0%
c. Both	16%
**Q5: What percentage of patients visited in the last month had:**	
a. % gastrointestinal tract tumors	39%
b. % pancreatic tumors	14%
c. % head and neck tumors	15%
d. % other (please, specify:::) (if 100%, quit the survey)	32%
**Q6: Your role is…**	
a. Director/Manager	18%
b. Chief medical oncologist	76%
c. Resident	1%
d. Other	5%
**Q7: How many beds are in the oncology department of your Center? (mean ± sd)**	25
**Q8: In what Italian geographical area do you work?**	
a. North-Western area	25%
b. North-Eastern area	17%
c. Center area and Sardinia	26%
d. South area and Sicily	32%
**Q9: How old are you? (mean ± sd; range)**	51 ± 9.5 years (range: 32–71)
**Q10: How frequently do you have to reduce the intensity/to stop the cancer therapy due to nutritional problems?**	
a. Never	5%
b. Rarely	47%
c. Sometimes	42%
d. Often	6%
**Q11.How frequently you have been unable to start the treatments due to patient**’**s malnutrition?**	
a. Never	7%
b. Rarely	40%
c. Sometimes	51%
d. Often	2%
**SECTION A: AWARENESS**	
**A1. Are you aware of the nutritional-metabolic problems that a cancer patient could experience?**	
a. yes	99%
b. no	1%
**A1.1. (if A1=a): What could be the problems?**	
**A2. Are you aware of the Guidelines for nutritional pathways in cancer patients issued by the Italian Ministry of Health in 2017?**	
a. yes	71%
b. no	29%
**A2.1 (if A2=a) In your opinion, the Guidelines have been properly implemented in the region where you work?**	
a. yes	46%
b. no	54%
**A3. Are you aware of the parallel nutritional-metabolic pathway for cancer patients, consisting in the development of an individual nutritional program associated to the oncological therapy since the first visit?**	
a. yes	85.5%
b. no	14.5%
**A4. Do you think that the parallel nutritional-metabolic pathway could be helpful for the improvement of the outcome in cancer patients?**	
a. yes, because::::::	99%
b. no, because::::::_	1%
**A5. The PreMiO study, an observational study performed in 22 medical oncology centers across Italy on 1952 patients at their first oncological visit, showed that 51% of patients were at risk for malnutrition and 9% of patients were overtly malnourished. In your opinion, to what extent these results could change the approach to nutritional screening?**	
a. very much	45%
b. sufficiently	47%
c. not much	6%
d. not at all	2%
**A6. Some evidences indicate an association between the depletion of lean mass and an increased risk for therapy toxicity. Do you think this association could be possible from a biological point of view? Is it in line with your experience?**	
a. Yes, because::::::_	95%
b. No, because::::::_	5%
**SECTION B: ATTITUDE AND PROPENSITY OF CLINICIANS AND THEIR CENTERS TOWARDS CLINICAL NUTRITION**	
**B1. In your opinion, to what extent the nutritional status affects the feasibility/tolerance of antineoplastic therapies in cancer patients?**	
a. very much	60%
b. sufficiently	37%
c. not much	2%
d. not at all	1%
**B1.1 To what extent do you agree with the following statements? Please use a 10-point scale, where 1 corresponds to “strongly disagree” and 10 corresponds to “strongly agree” (mean ± sd)**	
a. The malnutrition status of a cancer patient can negatively affect the antineoplastic therapies	9
b. If not adequately treated, the malnutrition status can negatively affect the antineoplastic therapies in a cancer patient	9.1
**B2. The ESPEN guidelines on nutrition in cancer patients, the results of the PreMiO study and the Guidelines for nutritional pathways in cancer patients issued by the Italian Ministry of Health push for a nutritional screening of all cancer patients at their first visit.** **What is your opinion?**	
a. I strongly agree	64%
b. I agree, but I would avoid a screening of all the patients	32%
c. I agree, I would screen all the patients, but not at the time of a diagnosis	4%
d. I disagree	0%
**B2.1 (if B2=c) If not at the time of a diagnosis, when would you screen all the patients?**	
**B3 What is the role of nutritionist for what concerns the cancer patients in your Center? (max two answers)**	
a. There is a specific protocol for the involvement of a nutritionist	24%
b. The nutritionist is involved on a case-by-case basis for specific patients	41%
c. The nutritionist is involved for patients identified through nutritional screenings	12%
d. The nutritionist is involved only for patients with advanced disease	2%
e. There is no intervention by a nutritionist	22%
B4. **Did your Center acknowledge and apply the Guidelines for nutritional pathways in cancer patients issued by the Italian Ministry of Health in 2017?**	
a. yes	77%
b. no	23%
**B4.1 (if B4=a) Did you notice any improvement? In case, what improvements?**	
**B4.2 (if B4=b) Why?**	
**B5. In addition to the Guidelines issued by the Ministry of Health, has your Center internal Guidelines and/or protocols for the administration of a nutritional therapy to cancer patients?**	
a. yes	71%
b. no	29%
**B6. What is the role of nutritional evaluation and support in management of cancer patients in your Center?**	
a. They are fundamental, as implemented in the treatment program	44%
b. They have a relevant role, but are performed occasionally	49%
c. They are not performed	7%
**B7 (if B6≠c) How the nutritional evaluation of cancer patients is performed in the Center where you work? (max two answers)**	
a. By validated screening tools (MUST, NRS2002, MNA, NRI, SGA)	38%
b. By instrumental and anthropometric measures	44%
c. By weight variations referred by the patient	16%
**B8 (if B6≠c) Who deals with screening/nutritional evaluation in the Center where you work? (please select all the answers that apply)**	
a. oncologist	29%
b. nutritionist	48%
c. nurse	6%
d. dietician	14%
e. other (please, specifiy:::::::::::::_)	2%
**B8.1 (if B6≠c) What patients are submitted to nutritional screening and evaluation? (please, select only one answer)**	
a. All the patients at their first visit	25%
b. Only some patients at their first visit	47%
c. All the patients when possible	11%
d. Only some patients when possible	16%
**B9. (if B6≠c) With reference to your experience, what type of nutritional support is most frequently prescribed to cancer patients in the Center where you work? (max two answers)**	
a. Nutritional counseling	31%
b. Use of oral supplements	43%
c. Artificial nutrition	24%
**B10. What percentage of body weight loss do you think should require the start of a nutritional therapy?**	
a. <5%	6.0%
b. from 5% to 10%	67.5%
c. >10%	26.5%
**B11. Is there a nutritional team in the Center where you work?**	
a. yes	46%
b. no	54%
**B11.1 (if B11=a) Who are the team components? (open-ended question)**	
**B11.2 Have you ever been part of a nutritional team?**	
a. yes	18%
b. no	82%
**B11.3 (if B11.2=b) Do you think an oncologist could be helpful as member of a nutritional team?**	
a. yes	81%
b. no	19%
**B12. Based on your experience, what cancer patients are most frequently directed towards artificial nutrition at home? (max two answers)**	
a. Patients submitted to active-therapeutic treatments	26%
b. Patients submitted to palliative care	45%
c. Patients submitted to surgery	27%
d. Other (please, specify::::::::::::::_)	2%
**B13. In your opinion, what should be the efficacy markers of the parallel nutritional-metabolic pathway? (please, select three in order of priority)** ::::::::::::	
**B14. In your opinion, what are the obstacles to the implementation of the parallel nutritional-metabolic pathway? (max three answers)**	
a. Shortage of time	34%
b. Lack of adequate personnel to this end	35%
c. Lack of an unanimous protocol	17%
d. Lack of sufficient evidences from controlled clinical trials	12%
e. Other (please, specify:::::::::_)	2%
**SECTION C: HOW TO INCREASE THE AWARENESS OF PHYSICIANS ABOUT THE IMPORTANCE OF CLINICAL NUTRITION IN THE ONCOLOGICAL SETTING**	
**C1. Despite the high prevalence and the clinical impact of malnutrition in cancer patients, the importance of a treatment seems to be still underestimated. How do you think this attitude could be explained? (max three answers)**	
a. Available evidences are insufficient	28%
b. The problem is real, but not clinically relevant	8%
c. Healthcare personnel training is insufficient	41%
d. It is an issue not sufficiently addressed in academic courses	21%
e. Other (please, specify:::::::::_)	2%
**C2. What would you suggest to increase the awareness about the clinical impact of malnutrition on cancer patients? (max three answers)**	
a. More information about the topic	34%
b. More research and support	19%
c. Creation of specific operational protocols	19%
d. Hiring more empolyees	28%
e. Other (please, specify::::::::::)	0%
**C3. Evidence in clinical practice indicates that nutritional treatments are reserved in particular to patients with advanced stage disease. What, among the following interventions, do you think could be more useful to anticipate the nutritional treatments to early stage disease? (Max one answer)**	
a. More evidences from controlled clinical studies	7%
b. More training	38%
c. Creation of organizational pathways aimed at treatment optimization based on available evidences	55%

Tools: The survey has been conducted through a mixed Computer-assisted Telephone Interviewing (CATI)/Computer-assisted web interviewing (CAWI) system. CATI is a surveying technique where an interviewer follows a script provided by a software application, while CAWI is a surveying technique in which the interviewee follows a script provided in a website, which is able to customize the flow of questions based on the answers provided. Both the techniques allow an efficient, standardized and optimized data collection, as the interviewer/interviewee enters the answers directly into the computer rather than sending a paper questionnaire for a subsequent acquisition of the data. Moreover, the questionnaires are administered in a more efficient and more accurate way, as the computer delivers the questions to the interviewer or to the interviewee in the right scheduled sequence. Finally, the CATI and CAWI techniques allow a more accurate data collection, the time required for collection of the answers is lower and data processing and follow-up are in real time. The questionnaire length was limited to 15 minutes.

### Statistical Analysis

Descriptive statistics were used to summarize participants’ demographic characteristics as well as the answers to survey questions. Pearson’s chi-squared test has been carried out and Kendall’s Tau correlation coefficient (Tau-b or Tau-c, range: −1; 1) has been calculated to measure the association(s) between the variables (i.e. geographical regions or different kinds of hospitals) and the answers to the survey. Eta coefficient was used when measuring the association between categorical (nominal, ordinal) and/or dichotomic variables. For all analyses, a p value < 0.05 was considered statistically significant. Data were analyzed using SPSS, version 26.0 (IBM SPSS, Chicago, IL, USA).

## Results

### Participant Characteristics

**Figure 1:**
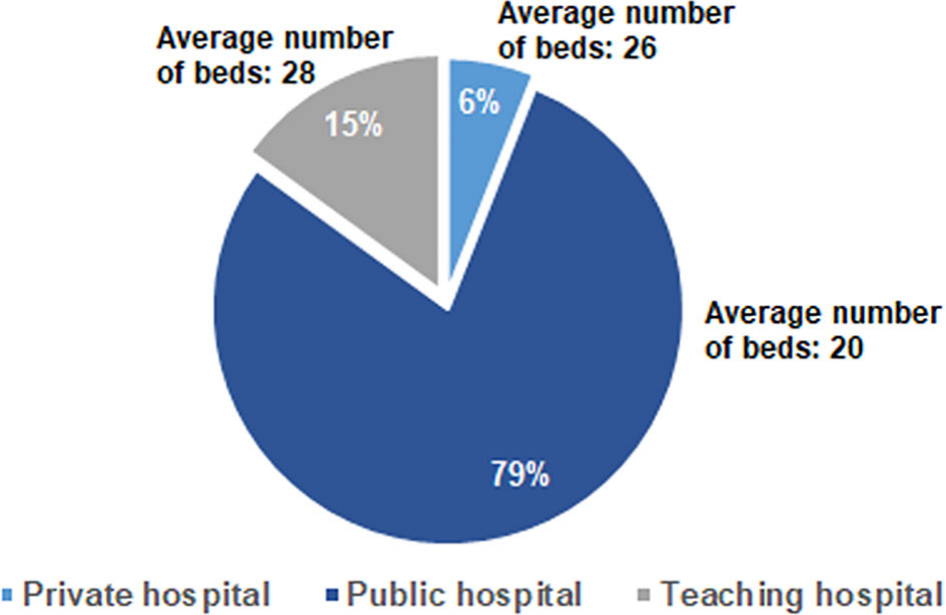
Distribution of the answers to the Q2 question: “Where do you work?” and to the Q7 question “How many beds are in the oncology department of your Center?”.

All the 300 invited medical oncologists participated to the survey and completed the questionnaire. The interviewees were equally distributed in different geographical regions in Italy and their mean age was 51 ± 9.5 years (range: 32–71). They worked mainly in public hospitals (79%) (**Figure 1**), with an average of 137 ± 89.3 patient visits/month and a focus mainly on solid tumors (84%). **Table 1** summarizes all the questions included in the survey and the answers, expressed as absolute values or as percentages.

### Awareness and Understanding of Malnutrition and Differences According to Geographical Areas

**Figure 2:**
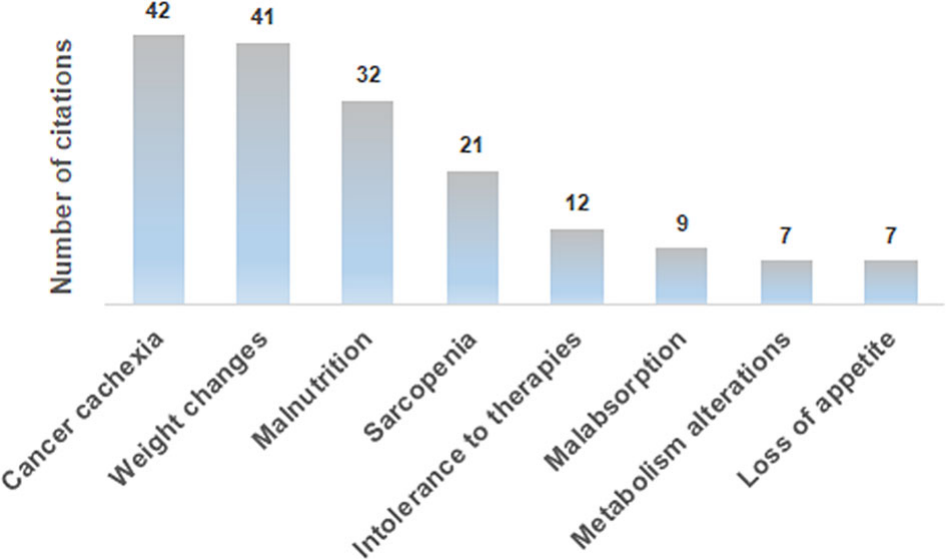
Most frequent answers to the A1.1 open-ended question: “What could be the nutritional-metabolic problems that a cancer patient could experience”?

The analysis of the awareness about the relevance of clinical nutrition in the oncological setting revealed that 51% and 42% of interviewees sometimes had been unable to start or had to reduce/stop a cancer therapy, respectively, due to patients’ nutritional problems. However, almost all the medical oncologists (99%) declared to be aware of the nutritional-metabolic problems that a cancer patient could experience, which mainly include, among others, cachexia, weight changes, malnutrition and sarcopenia (**Figure 2**). With reference to nutritional-metabolic problems of cancer patients, 71% of medical oncologists declared to be aware of the Guidelines for nutritional pathways in cancer patients issued by the Italian MoH in 2017 (15) that, however, according to 54% of interviewees, were not adequately implemented in reference geographical regions, but resulted to be acknowledged and applied in 77% of their centers. Despite about one third of the interviewees (29%) declared to be unaware of the Guidelines for nutritional pathways in cancer patients, 85.5% of them was aware of the parallel nutritional-metabolic pathway, declaring that it could be helpful to improve the disease outcome, as it positively affects the patient’s quality of life, in addition to his/her adherence and compliance to the therapy. Interestingly, the level of guidelines implementation (question A2.1, **Table 1**) was significantly different among geographical areas, being higher in the North of Italy (58.4%) with respect to both the Center (47.8%) and the South (30.4%) of the Country (p = 0.001, Eta = 0.246).

Where the guidelines have not been acknowledged, the interviewees stigmatize the lack of nutritionists or of dedicated healthcare professionals. The large majority of oncologists (92%) believe that the results from the PreMiO study (13) could sufficiently (47%) or deeply (45%) change the approach to nutritional screening. Of note, 95% of the interviewees agree with an association between the depletion of lean mass and an increased risk for therapy toxicity. In more detail, the answers to open-ended questions indicate that, according to survey participants, malnutrition in cancer patients could lead to cachexia and/or sarcopenia, in turn leading to depletion of lean mass and to a decreased immune response, with a consequent general body weakening and a lower drug metabolization. Impaired drug metabolism can increase treatment-related toxicity and thus reduce treatment tolerance. Other survey participants focused their attention on the greater protein waste related to lean mass depletion and to consequent metabolic changes.

### Attitude and Propensity of Clinicians and Their Centers Toward Clinical Nutrition

**Figure 3:**
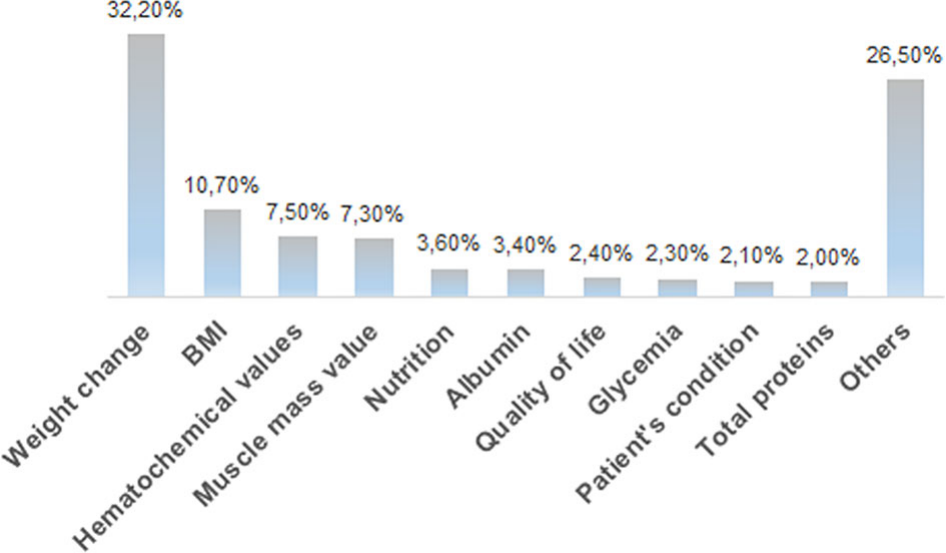
Distribution of the answers to the B13 multiple-choice question: “In your opinion, what should be the efficacy markers of the parallel nutritional-metabolic pathway”?

Medical oncologists agree that the nutritional status markedly affects the feasibility/tolerance of antineoplastic therapies in cancer patients (60%), and 64% of the interviewees fully agree with the need of a nutritional screening at the first oncological visit, as suggested by the ESPEN guidelines (14), by the MoH guidelines (15) and by the PreMiO study (13). Where the guidelines have been adopted, the oncologists observed a better tolerance to therapy. Despite this data, according to 41% of interviewees, the nutritionist is involved on a case-by-case basis for specific patients, while for 22% of survey participants there is no intervention by a nutritionist. Almost half of the medical oncologists (49%) indicate that nutritional evaluation and support in management of cancer patients have a relevant role, but these are carried out only occasionally, while in 44% of cases they are implemented in a treatment program. When they are carried out (by a medical nutritionist in 48% of cases and by a medical oncologist in 29% of cases), both instrumental and anthropometric measures are used in 44% of cases. Of major interest, only in 25% of cases all the patients are submitted to a nutritional screening and evaluation at their first visit. Oral supplements are the most frequently prescribed support (43%), followed by nutritional counseling (31%) and by artificial nutrition (enteral, parenteral) (24%). Home artificial nutrition is delivered in particular to palliative care (45%) and surgical (27%) patients. The efficacy indices of the parallel nutritional-metabolic pathway have been indicated by the participants to the survey through an open-ended question; weight changes, body mass index and blood test values obtained the highest priority (**Figure 3**).

#### Expert Review of Anticancer Therapy

Interestingly, statistically significant differences were observed regarding answers to questions B3 and B8.1 (**Table 1**) of the survey according to the geographical areas where the interviewees work. In particular, with reference to the question B3, the lack of intervention by a nutritionist (answer “e”, **Table 1**) was indicated by 29% of responders working in the South of Italy, 22% of responders working in the Center of Italy and 13% of responders working in the North of Italy (p = 0.005, Tau-c 0.134).

With reference to the question B8.1, the option “d”, indicating that only some patients are submitted to nutritional screening and evaluation when possible, was selected by 26% of responders working in the South of Italy, 15% of responders working in the Center of Italy and 6% of interviewees working in the North of Italy (p = 0.021, Tau-c 0,114).

Noteworthy, with reference to question B7, nutritional evaluation is assessed by validated screening tools (MUST, NRS2002, MNA, NRI, SGA) for 60% of responders working in teaching hospitals and 40.6% of responders working in public hospitals who selected this option (B7, “a”, **Table 1**), although this difference was not statistically significant (p = 0.286, Tau-b 0.133).

With reference to question B14 (**Table 1**), shortage of time was selected as an obstacle to the implementation of the parallel nutritional-metabolic pathway for 57.1% of interviewees working in private hospitals *vs.* 66.7% of interviewees working in teaching hospitals who selected this option (B14, “a”, **Table 1**) (p = 0.09, Tau-c 0.090).

### Suggestions on How to Increase the Awareness of Physicians About the Importance of Clinical Nutrition in the Oncological Setting

Almost half of the hospital oncologists involved in the survey ascribe the underestimation of nutritional treatments to the insufficient training of healthcare professionals. For this reason, the interviewees propose a greater information about the topic of malnutrition in cancer patients and hiring more employees, to increase the awareness about the impact of malnutrition in clinical practice. The majority of the hospital oncologists participating to the survey also supported the creation of organizational pathways aimed at treatment optimization based on available evidences, in order to anticipate the nutritional treatments to the early stage of the disease.

More than half of participants to the survey (54%) work in hospital centers where a nutritional team is absent, and, when present, it is composed mainly by a nutritionist (38%) or by a nutritionist and a dietician (28%). The large majority of interviewees had never been part of a nutritional team (82%), but they believe it could be helpful for a better management of patients. Shortage of time (34%) and lack of adequate personnel (35%) have been indicated as the main barriers to the implementation of a parallel nutritional-metabolic pathway in their Institution.

According to 41% the interviewees, the underestimation of the importance of a nutritional treatment in cancer patients can be explained by an insufficient training of healthcare personnel, which could be compensated by an increased information about the topic, as suggested by 34% of interviewees.

Finally, according to 55% of survey participants, the creation of organizational pathways aimed at treatment optimization could be useful to anticipate the nutritional treatment of cancer patients to early stage disease.

## Discussion

Weight loss and malnutrition in cancer patients are the result of inadequate nutritional balance due to several causes, including appetite loss, physical restrictions (e.g. pain, vomiting, ulcers), malabsorption, drug treatments, and are associated with worsened prognosis (18, 19). Numerous cytokines, including tumor necrosis factor-alpha, interleukin-1, interleukin-6, and interferon-gamma, released either by tumor cells or by the host as inflammatory reaction to tumor cells, have been postulated to play a role in the etiology of cancer cachexia. In particular, a massive release of cytokines can trigger effects that mimic the leptin hormonal signaling and suppress the ghrelin and neuropeptide Y signaling, without a compensatory response, thus inducing the sustained anorexia frequently observed in cancer patients (20).

It is widely accepted that malnutrition and cancer cachexia affect the outcome of oncological therapies and the patients’ quality of life (21–23). Studies focusing on different types of cancer highlighted a link between malnutrition/cachexia and worsening of treatment outcomes (24, 25). However, the importance of malnutrition and cancer cachexia is still underestimated *vs.* the cancer treatment, which remains the main focus in oncological clinical practice.

The results of the survey indicate that almost all the interviewees are aware of the nutritional-metabolic problems that a cancer patient could experience and of the importance of adequate nutrition during the oncological therapy. However, the data resulting from this survey highlight that nutritional support is not yet fully managed consistently with the available evidences and guidelines (13–15). The results we obtained are, at least in part, in agreement with the results from another survey among Italian oncology units and patients’ associations, showing that nutritional assessment and support were implemented into patients’ care since the diagnosis only for 35% of oncology unit referees. In addition, for 42% of oncologists, nutritional evaluation was performed only upon patients’ request (26). Of note, the 100% of invited medical oncologists participated to the survey we described here and completed the questionnaire *vs.* the 5.7% of participants to a previous exploratory national survey conducted by the Italian Society of Medical Oncology (AIOM) and the Italian Society of Artificial Nutrition and Metabolism (SINPE) in 2015, thus highlighting a current increased awareness and consideration of nutritional issues among Italian oncologists (27).

Of interest, based on a multinational analysis of real-world evidence, clinical nutrition in oncology results to be an unmet medical need also in France, where a diagnosis of malnutrition at first hospitalization occurs in 10% of patients, 13% are diagnosed later on, and 77% have no malnutrition diagnosis, and Germany, where only 16% of patients received home parenteral nutrition around 3 months before death, resulting in a longer survival (28). The lack of knowledge about clinical nutrition by medical oncologists emerged as a barrier to adequate nutrition therapy in a recent survey carried out in Turkey, suggesting the need of strategies to improve access to clinical nutrition education at both undergraduate and postgraduate level (29).

As anticipated above, a strength of the survey research we carried out is the high number of medical oncologists who agreed to participate (300) and the high response rate (100% of recruited participants). This was a quite surprising result, probably related to the high motivation and interest for the topic of the survey and for the mixed CATI/CAWI technique we used, compatible with the high workload of hospital medical oncologists. Another strength of this study is the uniform distribution of interviewees on the whole national territory, known to be characterized by a marked heterogeneity in terms of healthcare assistance and resources. Of note, significant associations have been identified among some answers and the geographical region where the interviewees work or, to a lesser extent, the type of hospital where the interviewees work, suggesting a heterogeneous landscape in Italy in the context of awareness of malnutrition in cancer patients and guidelines implementation.

Also, the research we summarized provides insights and possible solutions to increase the awareness of clinical nutrition in the oncological setting in Italy, including more specific training and information for healthcare personnel and the creation of organizational pathways to anticipate the nutritional treatments of cancer patients at the early stages of disease. In this setting, ESPEN also suggested an update of the educational/academic courses and issued a focused position paper underlying the importance of nutrition education in all medical schools, providing some clues for a successful implementation of clinical nutrition in the medical curriculum at academic centers (30). A recent study investigating the role played in Italy by regional and national policies on patient access to oral nutritional supplements (ONS), an efficacious and cost-effective method for treating malnutrition, revealed the absence of a clear relationship between regional policies in terms of cost reimbursement to patients and their access to ONS. These data further support the need of national and systematic activities to increase the awareness of the importance of malnutrition prevention among healthcare providers (31). Malnutrition increases the length of stay of hospitalized patients and implies extra hospitalization costs, both at individual and at national level. A narrative review by Khalatbari-Soltani S et al. revealed that in Europe patients’ malnutrition carries a considerable economic burden, with an annual cost up to 120 billion euros, and an additional cost ranging between 1640 and 5829 euros per hospitalized patient and an overall cost ranging between 2.1 and 10% of the national health expenditure (32). Additional financial/structural resources devoted to the healthcare system could increase the implementation of clinical nutrition in the oncological setting and would possibly result in a final positive balance in terms of decreased burden of the disease and improvement of the quality of life and prognosis for cancer patients, with an overall favorable effect on national health expenditure, as supported also by the results of a Cochrane systematic review of the literature (33).

The main limitation of this study is that the data we collected concern exclusively medical oncologists working in Italy; however, the comparison with the data resulting from surveys carried out in other Countries, as described above, reveals that malnutrition of cancer patients is a worldwide unmet medical need.

Cancer cachexia could be addressed in the next future not only by early and tailored metabolic and nutritional interventions, but also through a multimodal intervention based on a combination of nutritional and physical activity with a series of drugs and supplements currently under investigation, and potentially able to modulate appetite, anabolic effects, inflammation and muscle mass and function, as reviewed elsewhere (34). Of interest, dietary modification could be helpful not only for reduction of chachexia/malnutrition in cancer patients, but also as a complement to established therapies to enhance the cancer treatment (35). The psychological effects of cancer disease on patients’ behavior and attitude, including interest in food, are also of main importance and should be carefully addressed through adequate education and information by nutritionists and other professionals in team. Such education and counseling about the importance of nutrition during cancer treatment and the specific nutritional needs of cancer patients should also involve the patient’s family members, to increase their knowledge, awareness and support. In this setting, a survey investigating the self-perception of malnutrition in cancer patients has been developed and disseminated by the European Cancer Patient Coalition to its members in 10 Countries, including Italy (36). The survey revealed a generally low self-perception of malnutrition in cancer patients, despite the large majority of patients (85.1% in Italy) reported weight loss after cancer diagnosis. Of note, the term “cachexia” was generally unknown to respondents. Of interest, the feasibility of self-completion of a Patient-Generated Subjective Global Assessment Short Form by head and neck cancer patients has been assessed in Netherlands by Jager-Wittenaar H et al. (37), highlighting an increased awareness on malnutrition risk in patients that completed the form.

## Conclusion

The survey results indicate that malnutrition of cancer patients remains, at least in part, an unmet medical need. As a matter of fact, the progressively increased knowledge about the pathogenesis of cancer cachexia has not been accompanied by effective strategies aimed at modifying the approach to this syndrome. An early diagnosis of cancer cachexia could allow a prompt intervention and minimize its adverse impact on cancer prognosis and patient’s quality of life. Consequently, awareness of the importance of implementation of a nutritional, physical activity and psychological support to oncological care in hospital units through the creation of multidisciplinary teams and the application of relevant guidelines is of central importance. The results of the survey we described highlight the urgent need of strategies to be adopted for an early assessment of the nutritional status in cancer patients, and for the implementation of a parallel nutritional and metabolic approach. The differences observed among the geographical areas not only suggest that the access to nutritional care for cancer patients is far from being homogeneous, but also imply that the intensity of corrective measures (e.g. educational activities) should be different depending on the different Italian regions in order for more patients to benefit from optimal cancer care.

## Data Availability Statement

The raw data supporting the conclusions of this article will be made available by the authors, without undue reservation.

## Author Contributions

All authors listed have made a substantial, direct, and intellectual contribution to the work and approved it for publication.

## Funding

The survey was made possible thanks to an unrestricted educational grant provided by Fresenius Kabi Italia. Fresenius Kabi Italia did not interfere in the design and conduction of the study, in data analysis and study conclusions.

## Conflict of Interest

EC was employed by Medi-Pragma S.r.l. 

The remaining authors declare that the research was conducted in the absence of any commercial or financial relationships that could be construed as a potential conflict of interest.
